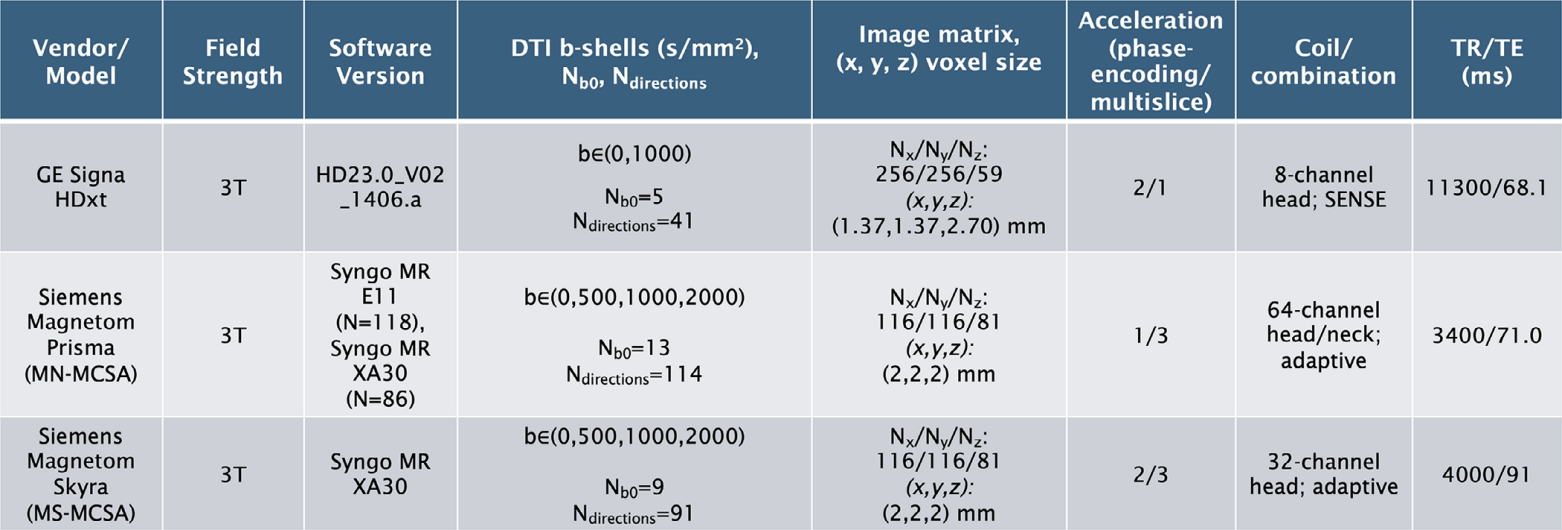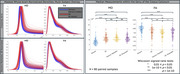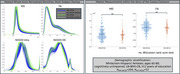# Diffusion MRI protocol standardization reduces measurement variability in the longitudinal Mayo Clinic Study of Aging

**DOI:** 10.1002/alz70862_109965

**Published:** 2025-12-23

**Authors:** Nolan K. Meyer, Robert I. Reid, Michael G. Kamykowski, Heather J. Wiste, Michael E. Griswold, B Gwen Windham, Arvin Arani, Kejal Kantarci, Jonathan Graff‐Radford, Ronald Petersen, Clifford R. Jack, Prashanthi Vemuri

**Affiliations:** ^1^ Mayo Clinic, Rochester, MN USA; ^2^ Department of Health Sciences Research, Mayo Clinic, Rochester, MN USA; ^3^ University of Mississippi Medical Center, The MIND Center, Jackson, MS USA; ^4^ Memory Impairment and Neurodegenerative Dementia Center, University of Mississippi Medical Center, Jackson, MS USA; ^5^ Department of Neurology, Mayo Clinic, Rochester, MN USA

## Abstract

**Background:**

Diffusion MRI (dMRI) measures are increasingly being used as biomarkers for aging and dementia in multi‐center studies. Measurements in longitudinal studies are often acquired on different systems, which can result in systematic differences of machine origin (e.g. vendor, hardware, algorithms). This may warrant harmonization prior to pooling multi‐site data. However, harmonization algorithms may introduce a risk of removing valuable information content and reducing sensitivity to physiological factors (e.g. sex, age, disease). Here, we tested the hypothesis that protocol standardization may minimize machine‐origin differences, obviating a need for post‐acquisition harmonization in a two‐site (Mississippi‐MS and Minnesota‐MN) cohort of the longitudinal Mayo Clinic Study of Aging (MCSA).

**Methods:**

Two data sets were analyzed: first, a crossover dataset with 80 participants (scanned within 2 days) bridging two different protocols on GE and Siemens scanners; second, participants scanned using standardized protocols from the ongoing MCSA (*N*_MN‐MCSA_=204; *N*_MS‐MCSA_=52). Parameters are listed in Figure 1. For crossover data, analyses were repeated with GE data resampled to 2‐mm isotropic resolution, and discarding Siemens‐only b‐shells to artificially reduce protocol disparities. Analysis encompassed visualization of overlain subjectwise histogram‐derived densities obtained from whole‐brain masks for dMRI measures; and median dMRI measures from within the genu of the corpus callosum (GCC) with nonparametric statistical testing. When available, both DTI and NODDI measures were compared.

**Results:**

Pronounced visual distributional differences seen between MD and FA in crossover data were reduced with resampling of GE data and including only b‐shells shared across acquisitions. For the resampled/reshelled data, significant differences remained for all GCC‐FA comparisons, but not all GCC‐MD (Figure 2). For the ongoing MCSA data from two sites, MD and FA whole‐brain densities were more closely aligned, with GCC‐FA and GCC‐MD showing no significant differences (Figure 3). NODDI measures showed more discordant densities than DTI measures, including shifts in most probable values and differences in distributional arrangement.

**Conclusions:**

Our work provides further evidence supporting protocol and processing standardization for minimizing machine‐origin differences prior to considering harmonization. Differences in dMRI metrics seen when comparing across unstandardized protocols were reduced when protocols were standardized. NODDI metrics had more apparent sensitivity to site than DTI measures.